# Insights into the involvement of long non-coding RNAs in doxorubicin resistance of cancer

**DOI:** 10.3389/fphar.2023.1243934

**Published:** 2023-09-15

**Authors:** Hai-Bo Zhang, Yang Hu, Jun-Li Deng, Guo-Ying Fang, Ying Zeng

**Affiliations:** ^1^ Department of Pharmacy, Hangzhou Women’s Hospital (Hangzhou Maternity and Child Health Care Hospital), Hangzhou, China; ^2^ Guangzhou Institute of Respiratory Disease and China State Key Laboratory of Respiratory Disease, The First Affiliated Hospital of Guangzhou Medical University, Guangzhou, China; ^3^ Department of Pharmacy, Zhujiang Hospital, Southern Medical University, Guangzhou, China; ^4^ Department of Pharmacy, The Affiliated Changsha Central Hospital, Hengyang Medical School, University of South China, Changsha, China

**Keywords:** long non-coding RNA, doxorubicin, drug resistance, cancer, molecular mechanisms

## Abstract

Doxorubicin is one of the most classical chemotherapeutic drugs for the treatment of cancer. However, resistance to the cytotoxic effects of doxorubicin in tumor cells remains a major obstacle. Aberrant expression of long non-coding RNAs (lncRNAs) has been associated with tumorigenesis and development via regulation of chromatin remodeling, transcription, and post-transcriptional processing. Emerging studies have also revealed that dysregulation of lncRNAs mediates the development of drug resistance through multiple molecules and pathways. In this review, we focus on the role and mechanism of lncRNAs in the progress of doxorubicin resistance in various cancers, which mainly include cellular drug transport, cell cycle disorder, anti-apoptosis, epithelial-mesenchymal transition, cancer stem cells, autophagy, tumor microenvironment, metabolic reprogramming and signaling pathways. This review is aimed to provide potential therapeutic targets for future cancer therapy, especially for the reversal of chemoresistance.

## Introduction

Cancer has become one of the most common diseases and a leading cause of death. It is estimated that 19.3 million new cancer cases occurred worldwide in 2020 with almost 10.0 million cancer deaths (Sung et al., 2021). In 2040, the global cancer burden is even expected to reach 28.4 million cases, representing a 47% rise compared to 2020 (Sung et al., 2021). Research on cancer has drawn extensive attention and great progress has been made regarding to cancer screening, early diagnosis and effective treatment. For example, the mortality rate of breast cancer (BRCA) is shown to fall steadily, with an about 35% decline over the past three decades (Malvezzi et al., 2019). Chemotherapy alongside surgery and radiotherapy, usually constitutes the standard regimen of cancer therapy (Mariette et al., 2007). However, when cancer is advanced or patients cannot suffer surgery, chemotherapy then becomes the last strategy.

Doxorubicin (DOX) is an anthracycline antibiotic which was isolated from the pigment-producing *Streptomyces* peucetius early in the 1960s (A et al., 2000). It is one of the most widely employed chemotherapeutic agents for the treatment of both hematological and solid tumors, including breast cancer, ovarian cancer (OC), bladder cancer (BLCA), lung cancer (LC), and acute myeloblastic leukemia (AML) (Blum and Carter, 1974; Young et al., 1981; Hulst et al., 2022). Doxorubicin stabilizes a reaction intermediate in which DNA strands are cut and covalently linked to tyrosine residues of topoisomerase II (Top2), eventually blocking DNA relegation (Minotti et al., 2004; Pommier et al., 2010). In addition, doxorubicin generates free radicals, leading to DNA damage or lipid peroxidation; interferes with DNA unwinding or DNA strand separation and helicase activity; induces apoptosis in response to Top2 inhibition (Gewirtz, 1999; Minotti et al., 2004; Pommier et al., 2010). DOX can also induce histone eviction from open chromatin, which attenuates the DNA damage response, triggers epigenetic alterations and induces apoptosis (Pang et al., 2013).

Since its discovery, DOX has brought a substantial improvement in cancer therapy. The introduction of DOX into the adjuvant therapy of BRCA demonstrated definite benefit in disease-free survival and overall survival (Hortobágyi and Buzdar, 1993). Gastric cancer (GC) was ever considered refractory to chemotherapy, whereas the addition of DOX produced encouraging response rates over 40% and increased median overall survival (Wadler et al., 1985). Along with the wide application, intrinsic and acquired resistance to DOX remains a major clinical problem. Some studies revealed resistance to DOX due to increase of drug efflux and reduction in drug accumulation, mediated by members of the ATP-binding cassette (ABC) superfamily (Grant et al., 1994; Velamakanni et al., 2007; Broxterman et al., 2009). The members of ABC transporters regulate the absorption, distribution, and clearance of pharmacological agents (Vasiliou et al., 2009). However, though many investigations are devoted to the development of transporter inhibitors for reversal of resistance, it has not been successful in improving the clinical response to chemotherapy, causing our consideration on the real nature of chemoresistance (Abraham et al., 2009). Undoubtedly, it will be of key importance for clinical studies to define the exact mechanisms mediating doxorubicin resistance.

Long non-coding RNAs (lncRNAs) are a kind of transcriptional products with a length longer than 200 nucleotides and no or low protein-encoding ability (Alexander et al., 2010; Uchida and Dimmeler, 2015; Yang L et al., 2014). Similar to coding genes, lncRNAs are usually transcribed by RNA polymerase II and have a poly-A tail (Gibb et al., 2011), but their sequence are less conserved than that of mRNAs (Pang et al., 2006). LncRNA has only been regarded as the “transcriptional noise” of the genome, rather than having biological functions, for a long time after its discovery (Ponjavic et al., 2007; Struhl, 2007). In recent years, more and more studies have shown that lncRNAs are widely involved in the regulation of gene expression at epigenetic, transcriptional and posttranscriptional levels (Yang Get al., 2014), playing an important role in cell differentiation, organogenesis, tissue homeostasis and other critical life activities (Mercer et al., 2009; Hung and Chang, 2010; Schmitz et al., 2016). In addition, the abnormal expression of lncRNAs is also closely related to the occurrence and development of cancer and chemoresistance (Batista and Chang, 2013; Evans et al., 2016). Insights into the role of lncRNAs in DOX resistance will help to deepen our understanding of chemoresistance formation and provide potentially targetable or predictive biomarkers of chemotherapy, which is the point of our review.

## lncRNA and doxorubicin resistance

**FIGURE 1:**
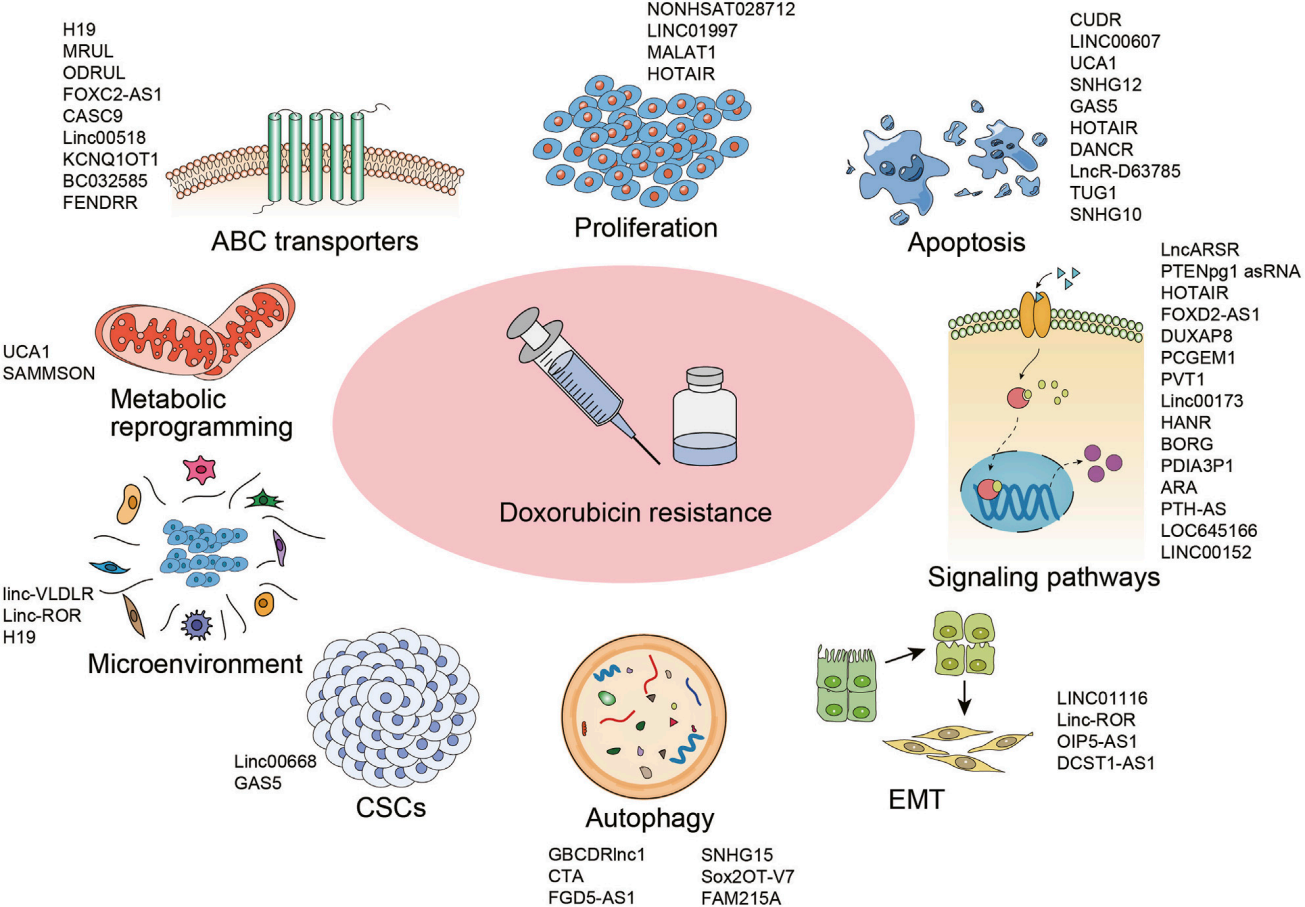
Mechanisms underlying doxorubicin resistance in cancer and the implicated lncRNAs. Abbreviations: ABC, ATP-binding cassette; EMT, epithelial to mesenchymal transition; CSCs, cancer stem cells.

LncRNAs have received extensive attention for its modulation in cancer progress as well as therapeutic response. Aiming to uncover the role and mechanism of lncRNAs in DOX resistance, databases were searched for published reports focused on “lncRNA and doxorubicin resistance”. The qualified articles were further straightened out and categorized in this section with the main mechanisms and implicated lncRNAs summarized in Figure 1.

## ABC transporters

**FIGURE 2:**
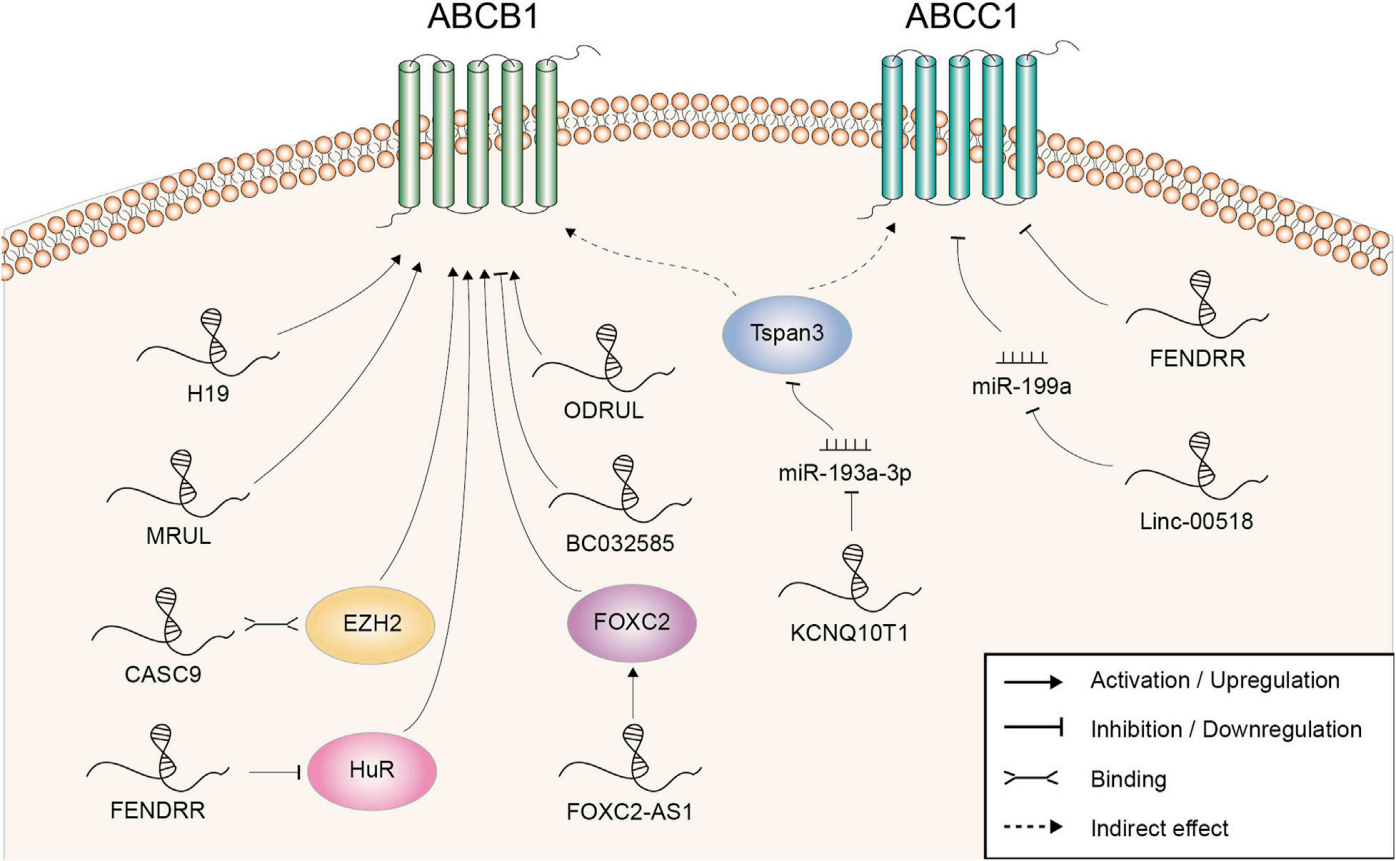
Doxorubicin resistance-related lncRNAs involved in the regulation of ABCB1 and ABCC1 and the underlying mechanisms.

The ATP-binding cassette (ABC) transporter family is a big family regulating cellular levels of hormones, lipids, ions, xenobiotics and other small molecules (Robey et al., 2018). Altered membrane transport and enhanced drug efflux mediated by over-expression of ABC superfamily, including ABCB1 and ABCC1, is one of the main and most studied mechanisms of doxorubicin resistance (Lehne, 2000). Known LncRNAs mediating the regulation of transporter expression were summarized in Figure 2.

H19 was the first lncRNA found to be implicated in ABCB1 regulation in human hepatocellular carcinoma (HCC). Reduced expression of H19 could suppress ABCB1 expression, which led to the increase of cellular DOX concentration and DOX sensitivity (Tsang and Kwok, 2007). Mechanistically, ABCB1 gene promoter was hypomethylated in resistant HCC cells, while H19 silencing induced a marked increase in ABCB1 promoter methylation and decrease in ABCB1 expression (Tsang and Kwok, 2007). There was also evidence that lncRNA MRUL could contribute to DOX resistance by playing an enhancer-like role in promoting ABCB1 expression in GC (Wang et al., 2014). MRUL knockdown led to increased drug accumulation and apoptosis in DOX-resistant SGC7901 cell (Wang et al., 2014). LncRNA microarray revealed that the expression levels of over 3,000 lncRNAs were altered in the DOX-resistant osteosarcoma (OSA) cell line MG63/DXR compared with the parental MG63 cell and ODRUL was the most upregulated one (Zhang et al., 2016). ODRUL might participate in DOX resistance by targeting ABCB1. In addition, the clinical results showed that high expression of ODRUL was correlated with poor chemotherapy response and prognosis (Zhang et al., 2016). Recently, Zhang et al. found that lncRNA FOXC2-AS1 expression was significantly higher in DOX-resistant OSA cell lines and tissues, and correlated with poor prognosis (Zhang C. L. et al., 2017). Functional studies revealed that silencing of FOXC2-AS1 abolished the growth of DOX-resistant OSA cell and improved the sensitivity to DOX *in vitro* and *in vivo*. Further mechanistic studies demonstrated that FOXC2-AS1 promoted the expression of transcription factor FOXC2 at both the transcription and post-transcription levels, further stimulating the expression of downstream ABCB1. CASC9, a lncRNA upregulated in doxorubicin-resistant BRCA cell, might regulate the expression of ABCB1 through EZH2. EZH2 was demonstrated to be a binding protein of CASC9. Meanwhile, EZH2 depletion resulted in suppressed ABCB1 expression (Jiang et al., 2018). Linc00518 and ABCC1 expression were both upregulated in DOX-resistant BRCA cell. Linc00518 could act as a molecular sponge of miR-199a to upregulate ABCC1 expression, thus conferring chemoresistance to DOX (Chang et al., 2018). LncRNA KCNQ1OT1 was upregulated in DOX resistant AML samples and cells. Through adsorbing miR-193a-3p, KCNQ1OT1 induced the expression of Tspan3. Unfortunately, the underlying mechanism of Tspan3 in chemoresistance was not revealed by the authors or reported elsewhere. However, the expression of ABCC1 and ABCB1 was found to be strictly regulated by Tspan3 (Sun et al., 2020).

Using the lncRNA expression profiling of BRCA patients from Gene Expression Omnibus datasets, our group screened out three lncRNAs (AK291479, U79293, and BC032585) to be significantly associated with anthracycline-based chemotherapeutic response (Zeng et al., 2019). BC032585 was further chosen to figure out its molecular function *in vitro*. It was observed that knockdown of BC032585 resulted in a stronger resistance to DOX as accessed by cell viability and this function was at least partly mediated by the upregulation of ABCB1. Collectively, this study had opened out a new approach for the identification of clinically useful lncRNA markers.

LncRNA could also negatively regulated ABCB1 expression and acted as a chemosensitivity mediator. LncRNA microarray found that FENDRR was the most downregulated lncRNA with a 22-fold change in the paired DOX-resistant and sensitive human OSA cell lines (Kun-Peng et al., 2017a). Functional studies revealed that FENDRR suppressed cell cycle, promoted apoptosis and increased DOX sensitivity of OSA cells *in vitro*. Moreover, further studies demonstrated that FENDRR inhibited DOX resistance through negatively affecting posttranscriptional expression of ABCB1 and ABCC1 (Kun-Peng et al., 2017b). FENDRR was also downregulated in resistant chronic myeloid leukaemia (CML) cells. The overexpression of FENDRR attenuated DOX resistance, as shown by increased DOX accumulation and enhanced cell apoptosis *in vitro* and *in vivo*. Both FENDRR and ABCB1 mRNA contained several AU-rich elements and competitively bound to the RNA-binding protein HuR (Zhang et al., 2019). Previous studies indicated that this interaction with RNA-binding protein was beneficial to keeping the mRNA stabilization and/or regulating the translation (Bish and Vogel, 2014). As a result, aberrations in FENDRR expression led to the opposite change of ABCB1 level.

## Apoptosis

**FIGURE 3:**
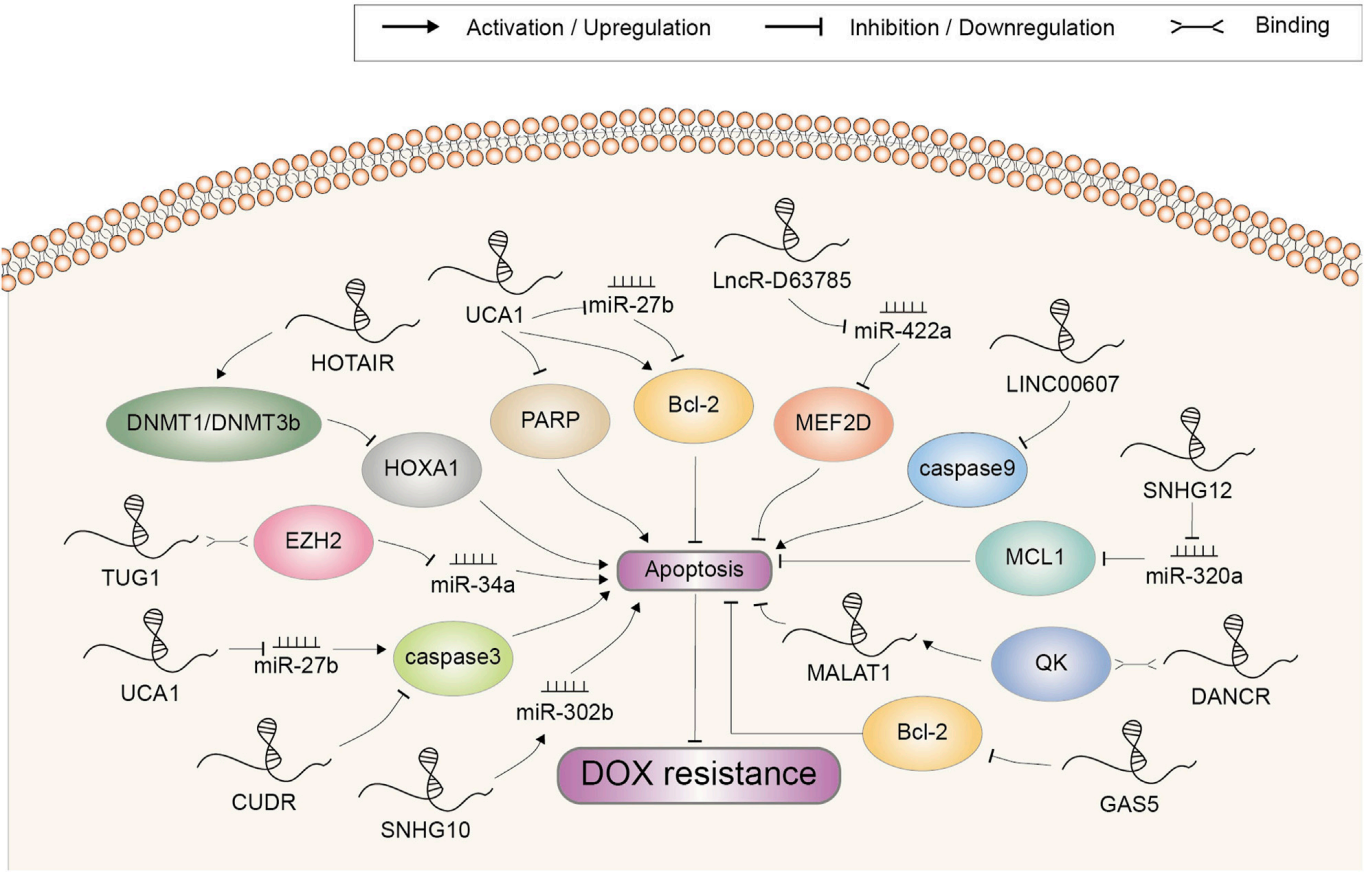
Doxorubicin resistance-related lncRNAs involved in the regulation of cell apoptosis and the underlying mechanisms. Abbreviations: DOX, doxorubicin.

In addition to targeting the multidrug transporter proteins, a part of lncRNAs involved in DOX resistance have been shown to regulate apoptosis-related genes (Figure 3). It is unsurprising because triggering apoptosis induction to eliminate malignant cells is exactly the way how most chemotherapeutic drugs work (Mohammad et al., 2015).

Caspases are evolutionarily conserved cysteine proteases with a well-defined role in apoptosis. Mammalian apoptotic caspases are generally divided into the initiators (caspase 2, 8, 9, and 10) and the effectors (caspase 3, 6, and 7), all of which must undergo proteolytic activation to execute their function (Shi, 2002; Van Opdenbosch and Lamkanfi, 2019). CUDR was a novel gene found to be overexpressed in A10A cell, a DOX-resistant subline of human squamous carcinoma (HSC) A431 cell (Tsang et al., 2007). Since the CUDR cDNA sequence contained no distinct open reading frames, it was inferred that CUDR possibly exerted its function as a long non-coding RNA. Further study indicated that the CUDR-inhibited apoptosis was at least dependent on downregulation of caspase 3 (Tsang et al., 2007). LINC00607 was upregulated in DOX-resistant thyroid cancer (TC) cell. It decreased caspase 9 expression by promoting the methylation of caspase 9 promoter, thereby inhibiting the apoptosis induction and augmenting DOX resistance (Li L. et al., 2021).

Above-mentioned initiator caspases activation can be mediated by anti-apoptosis protein Bcl-2-regulated pathway under cytotoxic drugs-induced cellular stress (Shalini et al., 2015). Shang et al. found that UCA1 silencing advanced cell apoptosis induced by DOX in GC cell through promoting cleaved PARP protein expression and depressing the expression of Bcl-2, indicating a promoting role in resistance development (Shang et al., 2016). Another study also demonstrated that UCA1 increased chemoresistance of GC cell via negatively regulating miR-27b. Mechanistically, UCA1 knockdown or miR-27b overexpression increased DOX-induced cell apoptosis by decreasing Bcl-2 protein expression and increasing cleaved caspase 3 (Fang Q. et al., 2016). High expression of SNHG12 was correlated with chemoresistance to DOX and a poor overall survival in OSA. In addition, a higher expression of SNHG12 was revealed in DOX-resistant cells compared to parental sensitive cells. SNHG12 mainly targeted miR-320a to upregulate MCL1, which has been reported to be a Bcl-2 family apoptosis regulator and exhibit a crucial function in suppressing cell apoptosis (Zhao et al., 2017; Zhou B. et al., 2018). Notably, lncRNA GAS5 was reported to inhibit rather than promote chemoresistance in bladder transitional cell carcinoma (BTCC). Overexpression of GAS5 promoted the induction of apoptosis by DOX and depressed Bcl-2 expression, whereas upregulated Bcl-2 largely reversed GAS5-induced sensitivity to DOX. Clinically, BTCC patients with lower level of GAS5 had a significantly worse disease free survival (Zhang H. et al., 2017). Altogether, these data confirmed that lncRNAs could affect the response of cancer to DOX according to their regulation pattern in Bcl-2 expression.

HOTAIR was upregulated in the DOX-resistant small cell lung cancer cell (SCLC). Depletion of HOTAIR increased drug sensitivity by enhancing cell apoptosis and decelerating cell cycle progression. Moreover, HOTAIR knockdown reduced HOXA1 methylation by decreasing DNMT1 and DNMT3b expression. Summarily, HOTAIR modulated chemotherapy resistance in SCLC by regulating HOXA1 methylation (Fang S. et al., 2016). DANCR was found to be suppressed by DOX in a high throughput screening in colorectal cancer (CC) cell. Via interacting with the RNA-binding protein QK, DANCR enhanced the RNA stability of MALAT1, which further mediate the suppressive function of DANCR on DOX-induced apoptosis (Xiong et al., 2021). This study established DANCR as an important repressor of apoptosis in CC. LncR-D63785 was highly expressed in GC tissues and cells. Knockdown of lncR-D63785 fostered the apoptosis of GC cells treated with DOX. It functioned as a sponge of miR-422a and promoted chemoresistance by blocking miR-422-dependent suppression of MEF2D (Zhou Z. et al., 2018). TUG1, a lncRNA upregulated in DOX-resistant AML tissues and cells, could epigenetically suppress miR-34a expression via recruiting EZH2 to its promoter. Either TUG1 knockdown or miR-34a overexpression remarkably facilitated cell chemosensitivity by enhancing DOX-induced apoptosis (Li Q. et al., 2019). LncRNA SNHG10 was downregulated in triple negative breast cancer (TNBC) cells after DOX treatment, and overexpression of SNHG10 significantly promoted DOX-induced apoptosis. Mechanism research showed that SNHG10 could inhibit the development of resistance to DOX by upregulating miR-302b through methylation modulation (Aini et al., 2022).

## Cell proliferation

**FIGURE 4:**
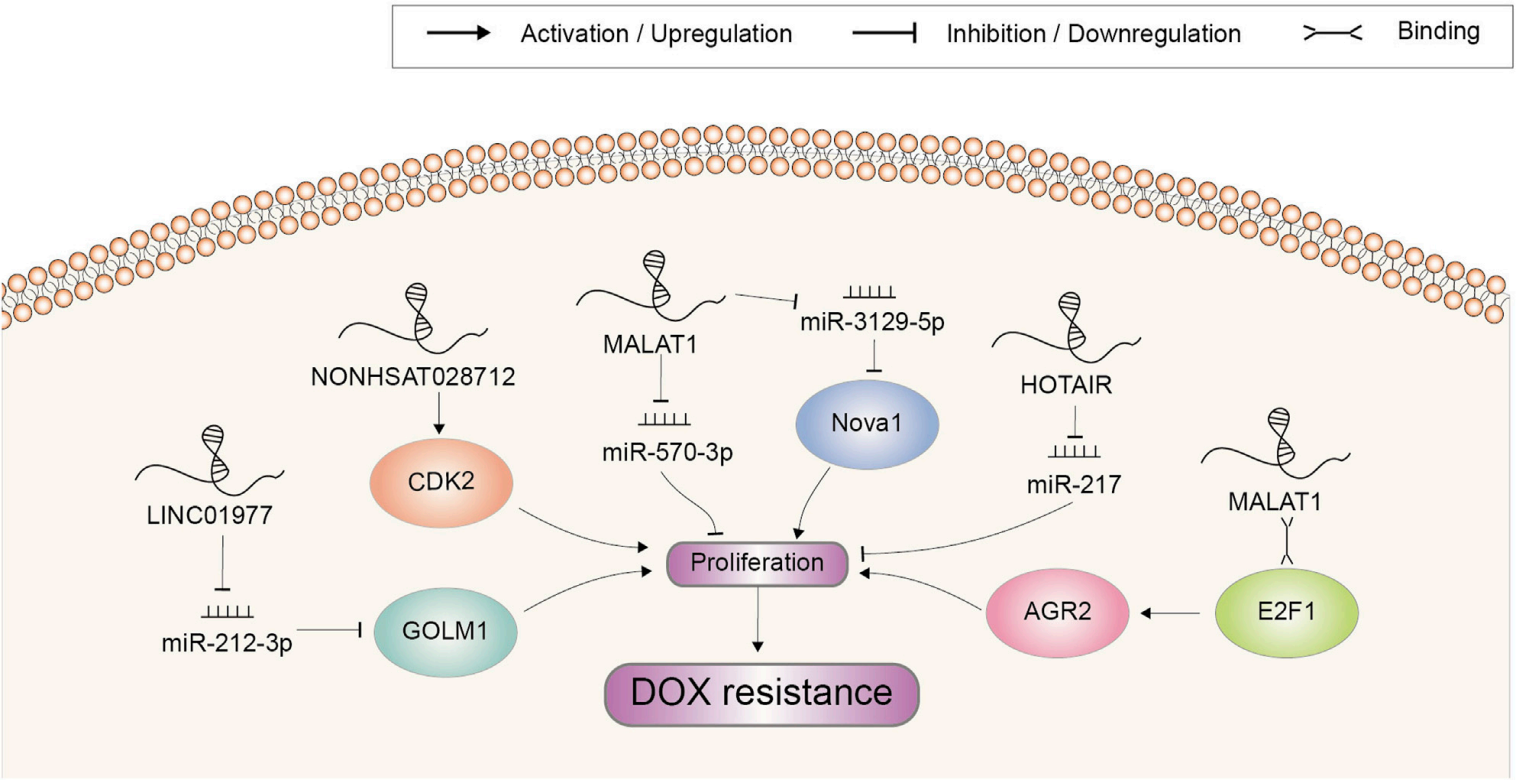
Doxorubicin resistance-related lncRNAs involved in the regulation of cell proliferation and the underlying mechanisms. Abbreviations: DOX, doxorubicin.

Uncontrolled proliferation is a hallmark of cancer, typically utilized by cancer cells to resist chemotherapeutic agent-induced growth suppression (Zheng et al., 2019). LncRNAs were also found to participate in aberrant cell proliferation and DOX resistance (Figure 4). Microarray analysis revealed that NONHSAT028712 was significantly increased in DOX-resistant BRCA cells. Further study indicated that NONHSAT028712 mediated the development of chemoresistance through cis-regulating nearby CDK2 gene, which was required for the transition of cell cycle from G1 to S phase (He et al., 2016). LINC01977 could significantly promote BRCA cell proliferation and chemoresistance to DOX in *vitro* assays (Li Z. et al., 2021). It sponged miR-212-3p to prevent miRNA-mediated repression of GOLM1, which was reported to function as a key promoter of cell proliferation in several cancer types (Chen et al., 2015; Xu et al., 2017). MALAT1 was reported to be highly expressed in DOX-resistant BRCA cells. It could promote cell proliferation and colony formation to increase DOX resistance, mechanistically through recruiting E2F1 and activating downstream AGR2 expression (Li S. et al., 2022). MiR-570-3p was another target of MALAT1, which could inhibit the proliferation of BRCA cells and mediate the regulatory role of MALAT1 on DOX resistance (Yue et al., 2021). In HCC, elevation of MALAT1 also mediated tumor growth and DOX resistance via a MALAT1/miR-3129-5p/Nova1 axis (Cao et al., 2021). Other lncRNAs enhancing DOX resistance through increasing cell proliferation included HOTAIR (Wang H. et al., 2018). In GC cells, HOTAIR mainly counteracted with miR-217 to inhibit its suppressing effect in DOX resistance (Wang H. et al., 2018).

## Signaling pathways

A fine-tuned regulation of signal transduction pathways is crucial for maintaining cellular and tissue homeostasis (SMA, 2020). Aberrant activation of oncogenic signaling pathways often lead to the transformation of normal cells to cancer cells with the acquirement of malignant phenotype (Palla et al., 2022). Many drugs with the ability of blocking dysregulated signaling pathways have been developed for cancer treatment. However, due to the crosstalk inside signaling network, awakening of alternative survival signaling pathways have become one dominating mechanism of chemoresistance (Dent et al., 2009). Similarly, any survival signaling pathway activated in response to toxic stress might also help cancer cells to escape DOX-based chemotherapy (Table 1).

**TABLE 1:** LncRNAs implicated in doxorubicin resistance of cancer through multiple signaling pathways.

LncRNA	Cancer type	Molecular mechanism	Role in DOX response	References
**PI3K signaling pathway**				
LncARSR	HCC	decreasing PTEN expression, activating PI3K pathway and NF-κB	Resistance	Li et al. (2017), Li et al. (2022b)
PTENpg1 asRNA	OSA	Promoting PTEN transcription and PTEN mRNA stability	Resistance	Johnsson et al. (2013)
HOTAIR	AML	Increasing the hypermethylation of *PTEN* promoter	Resistance	Zhou et al. (2021)
HOTAIR	BC	Increasing PI3K, AKT and mTOR phosphorylation	Resistance	Li et al. (2019b)
FOXD2-AS1	BC	Increasing PI3K and AKT phosphorylation	Resistance	Nong et al. (2021)
DUXAP8	B-ALL	Increasing PIK3CA expression through sponging miR-29a	Resistance	Zhang et al. (2022)
**P53 signaling pathway**				
PCGEM1	PC	Inhibiting the expression of p53 and p21^Waf1/Cip1^	Resistance	Fu et al. (2006)
PVT1	BLCA	Promoting p53 ubiquitination through MDM2/AURKB cascade	Resistance	Jiang et al. (2022)
**Wnt/β-catenin signaling pathway**				
Linc00173	SCLC	Sponging miR-218 to upregulate the expression of Etk, NDRG1 and GSKIP	Resistance	Zeng et al. (2020)
HANR	HCC	Binding to GSKIP for regulating the phosphorylation level of GSK3β	Resistance	Duffy et al. (2014), Xiao et al. (2017)
**NF-κB signaling pathway**				
BORG	TNBC	Binding to and activating RPA1	Resistance	Gooding et al. (2019)
PDIA3P1	HCC	Binding to miR-125a/b and miR-124 to upregulate TRAF6	Resistance	Xie et al. (2020)
**MAPK signaling pathway**				
ARA	HCC	Unkown	Resistance	Jiang et al. (2014a)
**JAK-STAT signaling pathway**				
PTH-AS	BC	Upregulating the expression level of STAT1	Resistance	Akimoto et al. (2022)
LOC645166	BC	Binding to and recruiting NF-κB to promote GATA3 transcription	Resistance	Zheng et al. (2020)
**Hippo signaling pathway**				
LINC00152	RB	Sponging miR-613 to positively regulate YAP1	Resistance	Lu et al. (2010)
**Keap1/Nrf2/ARE signaling pathway**				
PVT1	TNBC	Promoting the protein stability of Nrf2 by inhibiting the binding of Keap1 to Nrf2	Resistance	Luo et al. (2020)

Abbreviations: DOX, doxorubicin; HCC, hepatocellular carcinoma; OSA, osteosarcoma; AML, acute myeloblastic leukemia; BC, breast cancer; B-ALL, B-cell acute lymphoblastic leukemia; PC, prostate cancer; BLCA, bladder cancer; SCLC, small cell lung cancer cell; TNBC, triple negative breast cancer; RB, retinoblastoma.

## PI3K signaling pathway

PTEN tumor suppressor is a negative regulator of the PI3K/Akt pathway and is epigenetically silenced in multiple cancers (Álvarez-G et al., 2019). LncARSR overexpression inhibited DOX-induced cell apoptosis and enhanced DOX resistance in HCC while knockdown of lncARSR showed the opposite effects (Li et al., 2017). LncARSR decreased PTEN expression and activated the PI3K/Akt pathway. Furthermore, the effects of lncARSR on DOX resistance could be reversed by PTEN depletion or PI3K/Akt pathway inhibitors. Taken together, upregulated lncARSR promoted DOX resistance through activating the PTEN/PI3K pathway (Li et al., 2017). Subsequent study by Li et al. revealed that lncARSR further activated NF-κB in a PI3K pathway-dependent manner. NF-κB transactivated lncARSR through direct binding and activation of lncARSR promoter, forming a positive feedback regulatory loop among lncARSR, Akt and NF-κB. And this regulatory loop together promoted DOX resistance (Li Y. et al., 2022). LncRNA PTENpg1 regulated PTEN expression through sequestering numerous PTEN-targeting miRNAs. Moreover, two antisense RNA (asRNA) transcripts isoforms (α and β) were encoded from the *PTENpg1* locus (Johnsson et al., 2013). The α isoform epigenetically regulated PTEN transcription via localizing to the *PTEN* promoter and catalyzing the formation of H3K27me3, while the β isoform interacted with PTENpg1 as an RNA:RNA pairing and post transcriptionally affected PTEN production. Suppression of this asRNA isoforms-regulated network led to a clear induction of PTEN protein level and a concomitant downregulation of pAKT. As a result, the OSA cells were significantly sensitized to DOX (Johnsson et al., 2013). Unlike PTENpg1 asRNA transcripts, HOTAIR was reported to modulate PTEN expression by increasing the hypermethylation of its promoter locus, thus suppressing PTEN expression and conferring DOX resistance in AML (Zhou et al., 2021). HOTAIR was also reported to reinforce DOX resistance by promoting the phosphorylation of AKT and activating AKT/mTOR signaling pathway in BC (Li Z. et al., 2019). Other lncRNAs implicated in PI3K signaling pathway and DOX resistance included FOXD2-AS1 in BC and DUXAP8 in B-cell acute lymphoblastic leukemia (B-ALL), further uncovering the central role of PI3K pathway in cancer DOX resistance (Nong et al., 2021; Zhang et al., 2022).

## P53 signaling pathway

LncRNA PCGEM1 was specifically expressed in prostate tissue, and associated with prostate cancer (PC). The overexpression of PCGEM1 attenuated DOX-induced apoptosis in LNCaP cells (Fu et al., 2006). Moreover, the induction of p53 and p21^Waf1/Cip1^ due to DOX treatment was attenuated by PCGEM1 overexpression, as well as the protein levels of cleaved caspase 7 and cleaved PARP. These implied that PCGEM1 induced DOX resistance by inhibiting the function of p53-dependent apoptotic machinery (Fu et al., 2006). In BLCA cells, lncRNA PVT1 could interact with MDM2, promoting its expression and cascaded MDM2/AURKB-mediated p53 ubiquitination. Thus, p53 pathway-mediated tumor suppressor genes were suppressed, leading to elevated proliferation, invasion, and DOX resistance. Furthermore, addition of the MDM2 inhibitor Nutlin-3 could offset the increased DOX resistance induced by PVT1 overexpression, while overexpression of MDM2 or AURKB reversed PVT1 knockdown-induced sensitivity to DOX (Jiang et al., 2022).

## Wnt/β-catenin signaling pathway

Linc00173 was first shown to be associated with the clinical stages and chemotherapeutic responses in SCLC. Elevated Linc00173 enhanced chemoresistance and cancer progression by sponging miR-218 to upregulate Etk expression. NDRG1 and GSKIP were positively regulated by Etk, which further induced the accumulation of β-catenin in the nucleus and activated Wnt/β-catenin pathway (Zeng et al., 2020). LncRNA HANR was demonstrated to be upregulated in HCC patients and predict a poor survival. Knockdown of HANR markedly enhanced the chemosensitivity of HCC cell lines to DOX, while overexpression of HANR showed the opposite effects. It was found that HANR bound to GSKIP for regulating the phosphorylation level and activity of GSK3β (Xiao et al., 2017). As a downstream target of GSK3β, Wnt/β-catenin pathway was thought to correspondingly perform its oncogenic function and impair the therapeutic outcome of DOX (Duffy et al., 2014).

## NF-κB signaling pathway

NF-κB signaling pathway could be provoked by genotoxic agents-induced DNA damage, augmenting the transactivation of varieties of anti-apoptosis genes and subsequent chemoresistance of cancer cells (Taniguchi and Karin, 2018). LncRNA BORG was greatly induced within TNBC cells when subjected to chemotherapeutic stresses. It fostered the cell survival and rendered them resistant to the cytotoxic effects of DOX both *in vitro* and *in vivo*. This chemoresistant activity of BORG was contingent upon its binding to RPA1, as well as the concomitant stimulation of NF-κB signaling. Interestingly, the activation of NF-κB amplifies BORG expression, which further enhances NF-κB activation, forming a novel feed-forward NF-κB signaling loop (Gooding et al., 2019). LncRNA PDIA3P1 was upregulated in human HCC and associated with poorer recurrence-free survival. DOX treatment could also upregulate PDIA3P1 level by disrupting the binding of hMTR4 to PDIA3P1 and abrogating the subsequent hMTR4-mediated degradation. TRAF6 was ordinarily suppressed by miR-125a/b and miR-124, while upregulated PDIA3P1 could bind to miR-125a/b and miR-124 to relieve their repression on TRAF6, leading to the activation of NF-κB pathway and reduced DOX-triggered apoptosis (Xie et al., 2020).

## MAPK signaling pathway

Jiang et al. discovered a new upregulated lncRNA named ARA in DOX-resistant BC cells, the expression of which was further found to be significantly associated with DOX sensitivity in a panel of BC cells as well as HCC cells. Knockdown of ARA inhibited cell proliferation and migration, induced G2/M cell cycle arrest and cell death, which together contributed to DOX resistance reverse. To investigate the functional role of ARA, microarray transcriptomic analysis was performed and genes regulated by ARA were enriched in multiple KEGG pathways, among which MAPK signaling pathway was the most outstanding (Jiang M. et al., 2014).

## JAK-STAT signaling pathway

Emerging data indicate that JAK-STAT pathway confers cellular resistance to antitumor treatment with DNA-damaging agents, including DOX (Khodarev et al., 2012). Akimoto et al. reported that ectopic expression of lncRNA PTH-AS in BC cell T47D markedly upregulated the level of STAT1 and its downstream interferon-related DNA damage resistance signature (IRDS) genes (Akimoto et al., 2022). As expected, when treated with DOX at a relatively high concentration, T47D cells with forced PTH-AS expression exhibited a significant resistance to drug-induced inhibition. LncRNA LOC645166 was identified to be upregulated in DOX-resistant BC cells as well as tissues of nonresponsive patients. It strengthened the tolerance of breast cancer to DOX via binding and recruiting NF-κB to promote GATA3 transcription, further leading to the activation of STAT3 (Zheng et al., 2020). The NF-κB/GATA3/STAT3 signaling pathway provided a promising target for overcoming DOX resistance in breast cancer.

## Hippo signaling pathway

In human retinoblastoma (RB), LINC00152 was reported to boost DOX resistance by sponging miR-613 to positively regulate YAP1 (Wan et al., 2020). YAP1 is a downstream effector of the Hippo signaling pathway, which is widely recognized as an important regulator in both organ size control and tumorigenesis (Lu et al., 2010). In previous studies, YAP1 had also been reported to have an effect on cell sensitivity to 5-fluorouracil and docetaxcel in esophageal cancer (Song et al., 2015). Thus, Hippo signaling pathway might be another molecular cascade responsible for lncRNA-mediated chemoresistance in cancer cells.

## Keap1/Nrf2/ARE signaling pathway

Keap1/Nrf2 signaling pathway plays an important role in maintaining cellular redox balance (Buti et al., 2013). Aberrant activation of this pathway has frequently been detected in human cancers and is also related to resistance to chemotherapies in established cancers (Ge et al., 2017). It was revealed that PVT1 promoted the protein stability of Nrf2 by inhibiting the binding of Keap1 to Nrf2, which potentiated the resistance of TNBC cells to DOX (Luo et al., 2020).

## Epithelial to mesenchymal transition (EMT)

**FIGURE 5:**
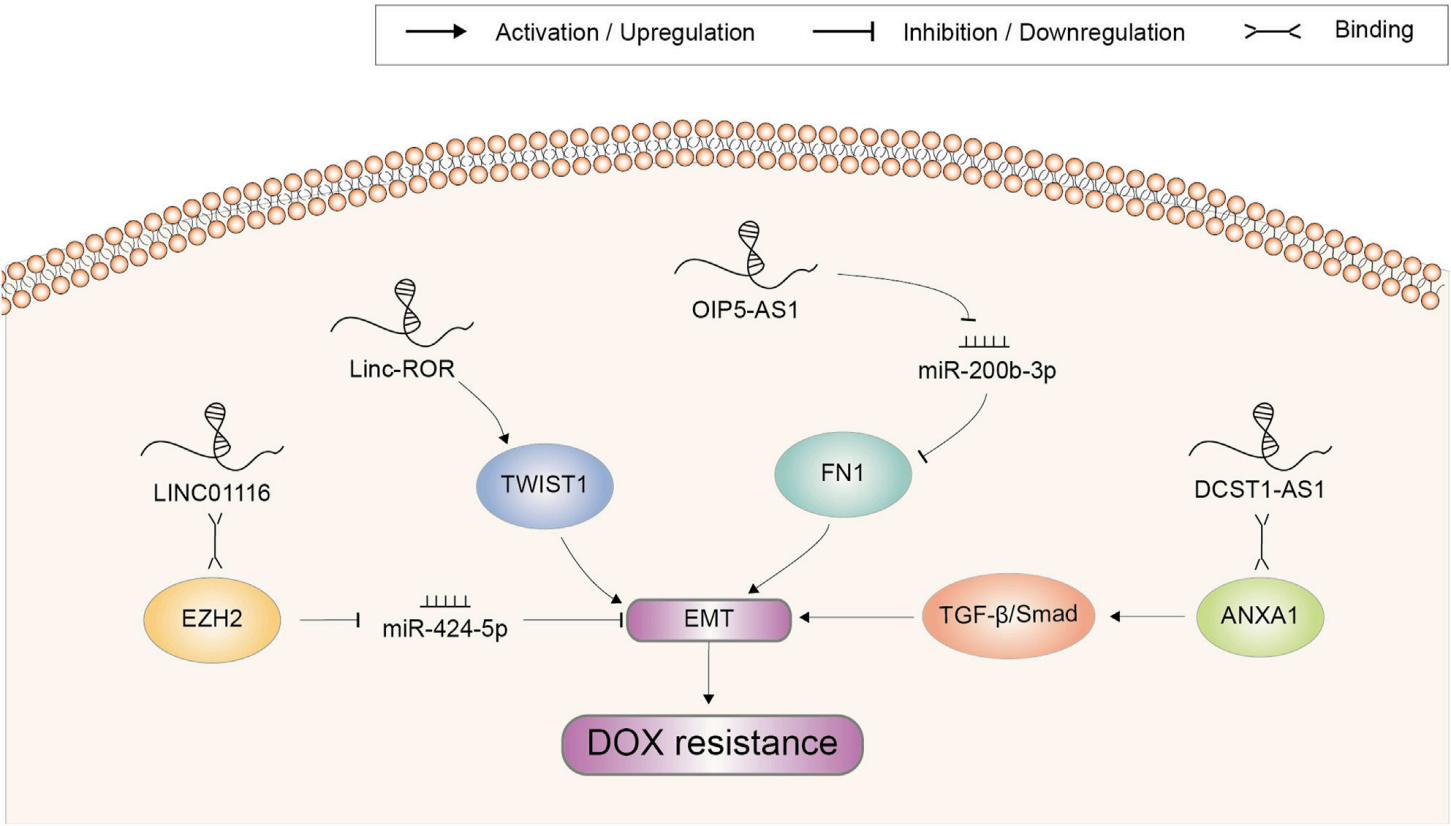
Doxorubicin resistance-related lncRNAs involved in the regulation of epithelial to mesenchymal transition and the underlying mechanisms. Abbreviations: DOX, doxorubicin; EMT, epithelial to mesenchymal transition.

EMT is a biological process in which epithelial cells transform into mesenchymal cells acquiring a motile phenotype (Pastushenko and Blanpain, 2019). Researches have revealed that EMT is not only closely related to tumor metastasis but also affects chemotherapy resistance. DOX resistance-related lncRNAs implicated in EMT regulation were summarized in Figure 5. In OSA, inhibition of LINC01116 suppressed cell viability, migration, and invasion, along with upregulated E-cadherin and downregulated vimentin. Accordingly, DOX resistance was attenuated. Further investigations indicated that LINC01116 regulated HMGA2 expression via EZH2-associated silencing of miR-424–5p and induced EMT (Li R. et al., 2021). Long intergenic non-protein coding RNA (linc)-regulator of reprogramming (ROR) was reported to promote invasion and metastasis in HCC. Knockdown of it notably suppressed EMT by downregulating TWIST1, increasing sensitivity of HCx`x`C cell to DOX (Zhang et al., 2021). FN1, is a glycoprotein present at the cell surface and in extracellular matrix tightly related to cellular adhesion and migration (Topalovski and Brekken, 2016). It was found to be significantly upregulated in the chemoresistant OSA cell lines and tissues and was related to unfavourable prognosis. LncRNA OIP5-AS1 acted as an upstream regulator of FN1 through sponging miR-200b-3p. Therefore, OIP5-AS1/miR-200b-3p/FN1 axis might be a promising target in treatment of OSA resistance to DOX (Kun-Peng et al., 2019). LncRNA DCST1-AS1 enhanced TGF-β/Smad signaling in TNBC cells through binding to ANXA1 and increasing its expression. Subsequently, the expression or secretion of proteins such as E-cadherin, SNAI1 and vimentin were coordinated to promote EMT and chemoresistance to DOX. Therefore, DCST1-AS1 represented a potentially promising therapy target for metastatic breast cancer (Tang et al., 2020).

## Autophagy

**FIGURE 6:**
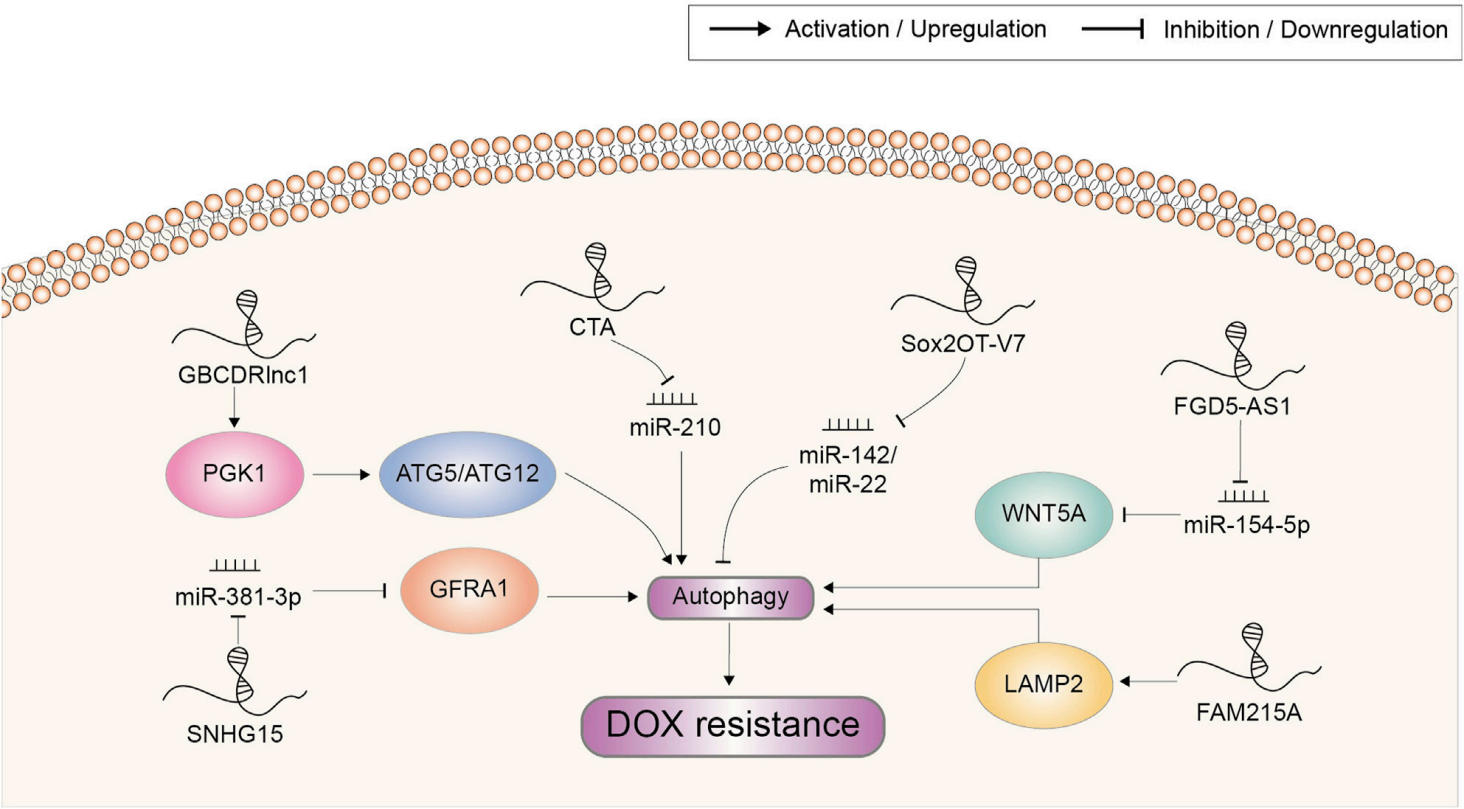
Doxorubicin resistance-related lncRNAs involved in the regulation of autophagy and the underlying mechanisms. Abbreviations: DOX, doxorubicin.

Accumulating evidence supported the cytoprotective role of autophagy in drug resistance of cancer. When under cytotoxic effects of chemotherapeutic drugs, autophagy could contribute to maintaining the intracellular homeostasis and prolonging the survival of cancer cells through autophagosomes (Carew et al., 2007). Recent researches have suggested that dysregulated lncRNA play a role in the development of chemoresistance via autophagy (Figure 6). For example, lncRNA GBCDRlnc1 served as a critical regulator of the autophagic activity and DOX-resistant property of gallbladder cancer. Through direct molecular interaction, GBCDRlnc1 prevented the ubiquitination of PGK1, leading to the upregulation of PGK1 protein level. The ATG5-ATG12 conjugate, an essential complex for autophagy initiation, might be a downstream target of the GBCDRlnc1/PGK1 axis. Knockdown of GBCDRlnc1 dramatically downregulated PGK1, ATG5 and ATG12, suppressed autophagy and improved the sensitivity of gallbladder cancer cells to DOX (Cai et al., 2019).

Wang et al. found that lncRNA CTA could be activated by DOX but was downregulated in DOX-resistant OSA cells. Overexpression of CTA could inhibit autophagy to overcome DOX resistance and promote apoptosis by competitively binding miR-210 (Wang et al., 2017). On the contrary, lncRNA FGD5-AS1 was upregulated in DOX-resistant OSA cells. It was reported to regulate the miR-154-5p/WNT5A axis by sponging miR-154-5p and thus potentiate autophagy-associated DOX resistance (Fei et al., 2022). Small nucleolar RNA host gene 15 (SNHG15) was also upregulated in DOX-resistant OSA cell lines. It elevated autophagy via targeting the miR-381-3p/GFRA1 axis to enhance DOX resistance (Zhang et al., 2020). Sox2OT-V7, another lncRNA involved in DOX resistance of OSA cells, could modulate autophagy through directly targeting miR-142/miR-22 (Zhu et al., 2020).

Lysosomes could sequester macromolecules generated from autophagy for degradation and recycling, and mediate multiple drug resistance in cancer (Piao and Amaravadi, 2016). In HCC, FAM215A was overexpressed and increased the resistance of cells to DOX-induced inhibition. This cell protective effect was proved to be achieved by stabilizing LAMP2, which constitutively contributed to lysosome formation and the maintenance of lysosomal content (Huang et al., 2020).

## Cancer stem cells (CSCs)

CSCs represent a small fraction of cells in the tumor featured by their potential of self-renewal and initiating tumors. Studies demonstrated that CSCs were responsible for chemoresistance and tumor recurrence following chemotherapy (Wicha et al., 2006).

Linc00668 was observed to be increased in BC compared to normal tissues. Forced expression of Linc00668 enhanced self-renewal capacity of BC cells as well as DOX resistance. Mechanistically, Linc00668 interacted with SND1 to augment its transcriptional activity and the expression of target genes, including Nanog, Sox2, and Oct4, which were critical regulators of stem cell-like properties (Qian et al., 2020). GAS5 was reported to function in maintaining stemness in human CC cell line HCT116-derived CSCs. GAS5 knockdown suppressed the self-renewal capacity of CSCs and sensitized them to DOX by inducing apoptosis. Moreover, inhibition of Nodal growth differentiation factor (NODAL) signaling presented the similar results. Therefore, it was hypothesized that GAS5 exerted protective effects in CSCs under DOX treatment in a NODAL signaling-dependent manner (Zhou and Xiao, 2020).

## Tumor microenvironment

Extracellular vesicles (EVs), mainly comprised of exosomes and microvesicles, are an important component of the tumor microenvironment (van Niel et al., 2018). EVs are a group of membrane-derived structures released by donor cell into the interstitial fluid. These EVs carry biological macromolecules such as protein, lipids and RNA, and can be taken up by recipient cells to achieve intercellular communication (Tkach and Théry, 2016). EVs are now considered as an additional mechanism for modulation of multiple physiological and pathological processes including chemoresistance (Mashouri et al., 2019).

Takahashi et al. (Takahashi et al., 2014a) identified a subset of lncRNAs in HCC that could be detected in EVs with at least 2-fold enrichment compared to donor cells. Among these lncRNAs, linc-VLDLR was also found to be significantly upregulated in malignant hepatocytes compared to non-malignant hepatocytes. Exposure of HCC cells to DOX increased linc-VLDLR expression within both cells and released EVs, and incubation with such EVs could reduce DOX-induced cell death in recipient cells. Further studies revealed that knockdown of linc-VLDLR suppressed cell viability, blocked cell-cycle progression and reduced the expression of ABCG2, leading to increased sensitivity of HCC cells to DOX (Takahashi et al., 2014a). Linc-ROR was another EV-transferred lncRNA in HCC (Takahashi et al., 2014b). Incubation with EVs originating from HCC cells increased linc-ROR expression and reduced DOX-induced cell death in recipient cells, whereas knockdown of linc-ROR augmented DOX-induced cytotoxicity. Besides, linc-ROR might mediate TGFβ-dependent chemoresistance in HCC, as TGFβ-increased expression of CD133+ tumor-initiating cells and colony growth were attenuated by linc-ROR knockdown. These findings all suggested an important role for linc-ROR in chemotherapeutic response of HCC (Takahashi et al., 2014b). LncRNA H19 had been proved to mediate the resistance of BC cells to DOX. Moreover, extracellular H19 could be incorporated into exosomes and delivered to sensitive cells, leading to the dissemination of DOX resistance. Therefore, exosomal H19 might be a potential target to reduce DOX resistance in BC (Wang et al., 2020).

## Metabolic reprogramming

Cancers have been shown to evade chemotherapy by switching to alternate metabolism. Aerobic glycolysis is recognized as an emerging hallmark of malignant tumors. Normal cells process glucose through mitochondrial oxidative phosphorylation, whereas glycolysis is preferred in most cancer cells for energy production, even under aerobic conditions (Hanahan and Weinberg, 2011). Moreover, emerging evidence has revealed that augmented glycolysis might also contribute to the development of acquired chemoresistance (Tamada et al., 2012). For example, the effect of UCA1 on DOX resistance in AML cell centered around its regulation of HIF-1α-dependent glycolysis. Ectopic expression of UCA1 exhibited a remarkable increase of glucose consumption and effectively enhanced HIF-1α level (Zhang et al., 2018). As a pivotal transcription factor, HIF-1α has been documented to play a critical role in metabolic reprogramming and chemoresistance in various tumor cells (Warfel and El-Deiry, 2014). LncRNA SAMMSON was overexpressed in DOX-resistant BC cell (Orre et al., 2021). Silencing of SAMMSON revealed a decreased glycolytic metabolism and an increased oxidative metabolism. Concomitantly, less ROS were produced from the mitochondrial respiratory chain, while mitochondrial replication, transcription and translation were enhanced. These results highlighted the role of SAMMSON in the metabolic rewiring and development of chemoresistance in BC.

## Targeting lncRNAs for reversing doxorubicin resistance

**FIGURE 7:**
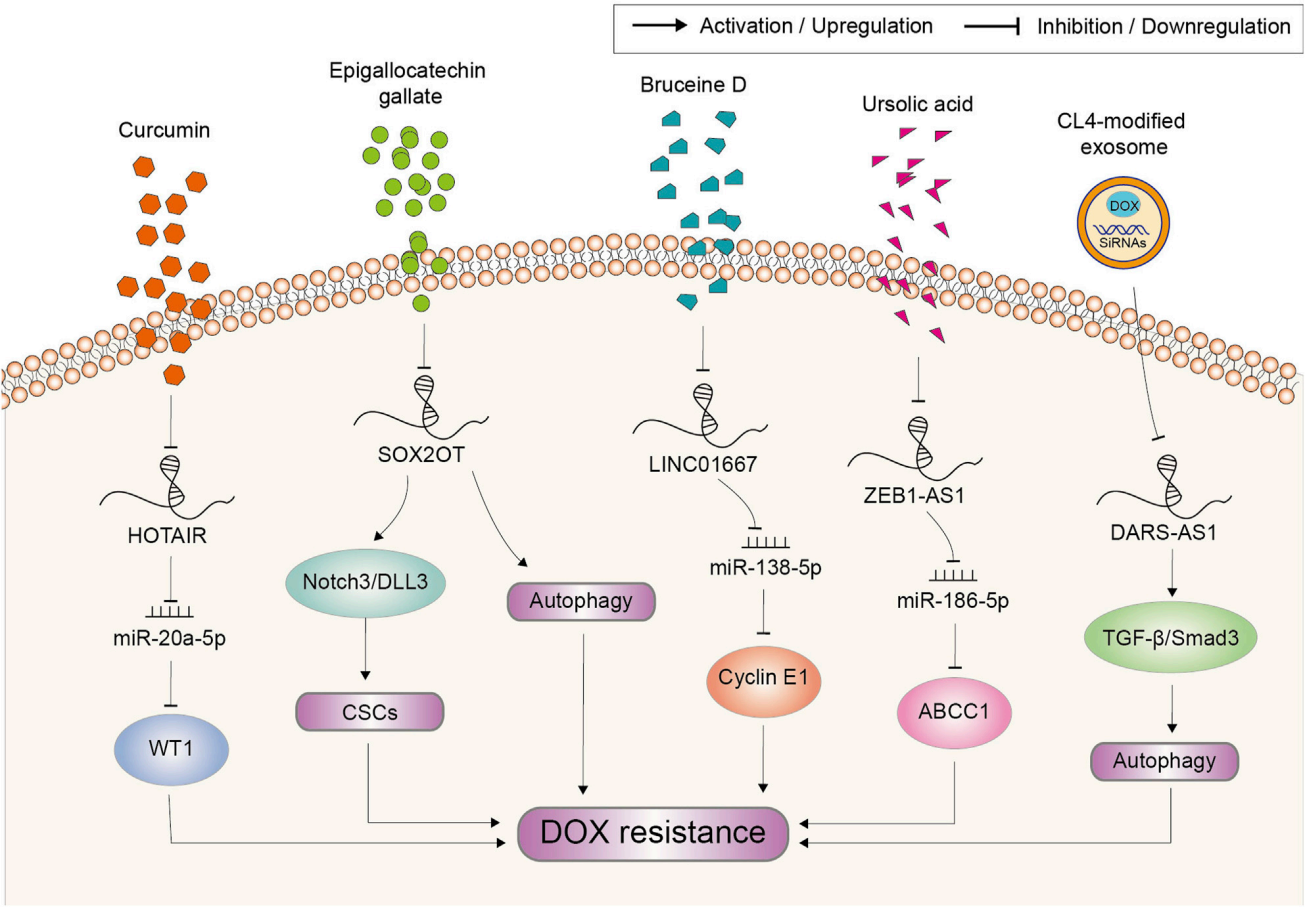
Potential strategies based on natural compounds and genetic tools to target lncRNAs for the reverse of doxorubicin resistance in cancer. Abbreviations: DOX, doxorubicin; CSCs, cancer stem cells.

Natural compounds including DOX occupy an important position in cancer therapy because of their diversity in structure and biological activity (Newman and Cragg, 2012). Owe to the multi-targeting capability, lncRNA might also be one of the targets of natural compounds. The recent reports regarding lncRNA-targeting natural compounds involved in DOX resistance are shown in Figure 7. Curcumin is a main active flavonoid component existing in Chinese herb *Curcuma longa* with the anti-tumor property (Moradi-Marjaneh et al., 2018). It had also been proved to suppress the resistance to DOX in acute myeloid leukemia. Mechanism study showed that lncRNA HOTAIR was inhibited by curcumin, which further mediated the sensitization effect of curcumin through the miR-20a-5p/WT1 axis (Liu et al., 2021). Epigallocatechin gallate is the highest content of polyphenol in green tea, which was reported to exert significant inhibitory effect on osteosarcoma cells including induce apoptosis, inhibit cell proliferation and invasion (Jiang L. et al., 2014). Moreover, Wang et al. reported that epigallocatechin gallate could produce synergistic effects with DOX on osteosarcoma cells by targeting lncRNA SOX2OT variant 7. On the one hand, epigallocatechin gallate decreased SOX2OT variant 7 to reduce DOX-induced autophagy, which played a pro-survival role in protecting cells from the growth inhibition of DOX. On the other hand, epigallocatechin gallate targeting SOX2OT variant 7 could partially inactivate the Notch3/DLL3 signaling cascade to reduce cell stemness then abate DOX resistance (Wang W. et al., 2018). Bruceine D (BD) is a quassinoid extracted from *Brucea javanica* which has an anti-tumor activity in various cancers (Lau et al., 2009). BD treatment in GC cells significantly downregulated the expression of LINC01667, further inhibiting the expression of Cyclin E1 by releasing miR-138-5p from LINC01667 sponge (Li et al., 2020). Thus, BD could inhibit the growth of GC cells and enhance the chemosensitivity of GC cells to DOX. Ursolic acid (UA), a pentacyclic triterpenoid compound, was reported to reverse DOX resistance in TNBC. It could inhibit the expression of ZEB1-AS1, which sponged miR-186-5p to upregulate ABCC1. Hence, UA treatment led to the decrease in ABCC1 expression (Lu et al., 2022). Together, combined therapy of above natural compounds with DOX might serve as an effective strategy to reduce the occurrence of chemoresistance and improve the curative effect in certain cancer, which needs further verification in the clinical practice.

Cancer occurs as a result of loss function of suppressor genes and activation of oncogenes (Caspi et al., 2021; Megyesfalvi et al., 2023). However, the conventional therapeutic using natural compounds or their analogs always lacks specific targets and induces serious side effects. Consequently, much attention has been directed towards the application of genetic tools in anticancer therapy. Small interfering RNA (siRNA) is the most extensively used tool applied in cancer therapy in the *in vitro* and *in vivo* study because of its potential in suppressing oncogenes (Mirzaei et al., 2021). As such tumor-promoting factors account for chemoresistance, targeting them through siRNA also provides an important strategy to reverse DOX resistance. However, the translational application of siRNA is still at its initial stage. There exist multiple limitations that challenge its efficacy, mainly including instability in blood circulation and incapability to enter cells (Ashrafizadeh et al., 2020). To overcome these difficulties, a variety of platforms have been developed for siRNAs delivery, which consist of lipid nanoparticles, liposomal nanoparticles, polymeric nanoparticles, silicon dioxide nanoparticles, carbon nanotubes, gold nanoparticles, iron oxide nanoparticles, aptamers and so on (Acharya et al., 2017). Nowadays, attempts based on these delivery platforms to target lncRNAs is still quite rare. A most recent study employed aptamer CL4-modified exosomes for the targeted delivery of DARS-AS1 siRNA and DOX to TNBC cells (Liu et al., 2023). The tumor growth was synergistically suppressed *in vivo*, while the delivery system did not induce any observed safety issues in mice. Meanwhile, *in vitro* experiments revealed that silencing DARS-AS1 decreased DOX resistance by suppressing autophagy via inhibition of the TGF-β/Smad3 signaling pathway (Figure 7). This study shows the outstanding application potential of genetic tool represented by siRNA in anti-cancer therapy and chemoresistance reverse.

## Conclusion and perspectives

Resistance to therapeutic drugs represented by DOX is a major burden for successful cancer treatments. However, the underlying mechanisms of chemoresistance are not yet fully elucidated. Multiple reasons for DOX resistance have been summarized and listed here, mainly including cellular drug transport, cell cycle disorders, anti-apoptosis, epithelial-mesenchymal transition, cancer stem cells, autophagy, tumor microenvironment, metabolic reprogramming and oncogenic signaling pathways. It should be noticed that cancer might develop resistance to DOX through more than one mechanism. What’s more, recently found hallmarks, such as altered metabolic reprogramming and tumor microenvironment (Hanahan and Weinberg, 2011), have not only affected the development of new means to treat human cancer, but also enriched the connotation of chemoresistance. Future study on the nature of cancer is still in urgent need and will undoubtedly provide direction for deepening our understanding of how chemoresistance develops.

At present, most of the reported lncRNAs associated with DOX resistance were identified from laboratory-based results, which were far away from clinical status. This might explain why the clinical translation of chemoresistance reversal is difficult. For example, after the discovery of ABCB1, a number of inhibitors were identified and added to chemotherapy regimens. However, they all failed in clinical trials due to the inefficiency or unbearable toxicity (Gottesman et al., 2002; Binkhathlan and Lavasanifar, 2013). Therefore, future work should be focused on identifying target lncRNAs through well-designed clinical approaches. Obtainment of matched pre- and post-progression tumor biopsies from patients with acquired DOX resistance would be of great importance.

Although the study of lncRNAs on chemoresistance is in its infancy, growing evidence suggests that lncRNAs may serve as potential molecular targets for cancer therapy as well as reversal of chemoresistance. Still, the method to target lncRNAs *in vivo* remains an unsolved problem. Compounds such as curcumin and epigallocatechin gallate can regulate lncRNA expression, but they are lack of specificity. To take lncRNAs as novel therapy targets, there is still a long way to go. Nevertheless, studies over the last decades have established a solid foundation to warrant further investigation of lncRNAs on reversing chemoresistance.
